# Perceived stress and emotional social support among women who are denied or receive abortions in the United States: a prospective cohort study

**DOI:** 10.1186/1472-6874-14-76

**Published:** 2014-06-19

**Authors:** Laura F Harris, Sarah CM Roberts, M Antonia Biggs, Corinne H Rocca, Diana Greene Foster

**Affiliations:** 1 University of California Berkeley, University of California San Francisco, Joint Medical Program, 570 University Hall #1190, Berkeley, CA 94720, USA; 2 Advancing New Standards in Reproductive Health (ANSIRH), University of California San Francisco, 1330 Broadway, Suite 1100, Oakland, CA 94612, USA

**Keywords:** Pregnancy, Abortion, Childbearing, Perceived stress, Social support

## Abstract

**Background:**

Examining women’s stress and social support following denial and receipt of abortion furthers understanding of the effects of unwanted childbearing and abortion on women’s well-being. This study investigated perceived stress and emotional social support over time among women who were denied wanted abortions and who received abortions, and compared outcomes between the groups.

**Methods:**

The Turnaway Study is a prospective cohort study of women who sought abortions at 30 abortion facilities across the United States, and follows women via semiannual phone interviews for five years. Participants include 956 English or Spanish speaking women aged 15 and over who sought abortions between 2008 and 2010 and whose gestation in pregnancy fit one of three groups: women who presented up to three weeks beyond a facility’s gestational age limit and were denied an abortion; women presenting within two weeks below the limit who received an abortion; and women who received a first trimester abortion. The outcomes were modified versions of the Perceived Stress Scale and the Multidimensional Scale of Perceived Social Support. Longitudinal mixed effects models were used to assess differences in outcomes between study groups over 30 months.

**Results:**

Women denied abortions initially had higher perceived stress than women receiving abortions near gestational age limits (1.0 unit difference on 0-16 scale, P = 0.003). Women receiving first-trimester abortions initially had lower perceived stress than women receiving abortions near gestational age limits (0.6 difference, P = 0.045). By six months, all groups’ levels of perceived stress were similar, and levels remained similar through 30 months. Emotional social support scores did not differ among women receiving abortions near gestational limits versus women denied abortions or women having first trimester abortions initially or over time.

**Conclusions:**

Soon after being denied abortions, women experienced higher perceived stress than women who received abortions. The study found no longer-term differences in perceived stress or emotional social support between women who received versus were denied abortions.

## Background

Perceived stress and lack of social support are psychosocial precursors to a range of mental and physical health problems [1-5]. The effects of denial versus receipt of abortion on women’s perceptions of stress and social support have not been studied. Having an abortion is a common experience among women in the United States [6]. Some argue that abortion is harmful to women’s well-being, citing increased risk of anxiety, depression, and suicide [7,8]. These claims are not supported by evidence from rigorous research [9-12] but little is known about the specific outcomes perceived stress and social support [13-15]. Understanding aspects of psychosocial well-being, such as stress and social support, among women with unwanted pregnancies who are unable to obtain an abortion is essential in order to plan for services to support these women.

### Perceived stress

Perceived stress is an individual’s global appraisal of the degree to which situations in her life are overwhelming [1]. It is a precursor for numerous poor health outcomes including inflammation and cardiovascular disease, and is a predictor of all-cause mortality [1,3,4,16].

Perceived stress during pregnancy is especially problematic because stress is associated with negative outcomes for both the pregnant woman and the baby, including low birthweight and premature birth; neonatal health issues, including impaired cognitive development; postpartum depression; and maternal-newborn attachment issues [17-20]. There is some evidence that women with unwanted pregnancies have higher perceived stress during pregnancy than women with wanted or mistimed pregnancies [21]. The postpartum period can also be a stressful time for women [22].

Some researchers have argued that abortion is stressful for women [7,8,23]. Results from rigorous research suggest that women’s psychosocial responses to abortion are not uniform and involve positive and negative emotions, including stress [9,12,13,24-26].

Although stress has been studied among women having abortions and among pregnant women, there has been no comparison of perceived stress between women with unwanted pregnancies who have abortions and those who deliver. In addition to the importance of understanding the experiences after each outcome, the comparison is significant because giving birth represents what would have happened to a woman if she were unable to terminate the unwanted pregnancy.

### Social support

A large body of evidence shows that social support – the receipt of resources, information or emotional caring through personal relationships – improves physical and mental health [5,14,19,27]. Social support is associated with better health during pregnancy and the postpartum period [28,29], and with reduced depression among new mothers, abortion patients, and other groups [13,14,30]. There is evidence that social networks change during the transition to parenthood [31]. Effective nation-wide programs such as the Nurse Family Partnership are based on the model that social support for low education, low income and/or unmarried pregnant women and new mothers improves long-term health and well-being for the mother and family [32].

Emotional social support – feeling that one is cared about – has been found to be strongly and consistently associated with good health and well-being [33]. A study of women who experience emotional difficulty after abortion found that lack of emotional support was a key reason for their negative feelings [34]. Emotional social support has been found to be associated with reduced odds of negative emotional response after an abortion [25]. Studies of women in the postpartum period have emphasized that emotional and instrumental social support are particularly important during this time [19,35,36]. Thus, emotional social support is important to consider when comparing receipt versus denial of abortion because low emotional support could contribute to development of health problems.

This analysis assesses whether receipt versus denial of abortion, as well as the gestational timing of the abortion, affects perceived stress and emotional social support both initially and over the two and a half years after seeking abortion. The study uses a prospective cohort design, which overcomes many of the weaknesses common to research on the effects of abortion such as underreporting of abortions, recall bias, inadequate control of confounders, and inadequate information on whether the outcomes of interest occurred after the abortion [9,10]. In addition, the longitudinal design allows for examination of trends over time.

## Methods

### Data source and collection

Turnaway Study design and recruitment, and some results, have been published elsewhere [25,37-42]. Briefly, participants were recruited between January 2008 and December 2010 from 30 abortion facilities across the United States. The study facilities had the latest gestational age limit for providing abortion care within a 150-mile radius; these limits ranged from 10 weeks to the end of the second trimester.

Eligible women were Spanish or English speakers 15 years or older with no fetal anomaly or demise who presented for abortion care. Women were recruited into one of three study groups: *Turnaway Group* – women presenting up to three weeks above the facility’s gestational limit who were denied an abortion; *Near Limit Abortion Group* – women within two weeks below the facility’s gestational limit who received an abortion; and *First Trimester Abortion Group* – women who received a first trimester abortion. Women were recruited into these groups in a 1:2:1 ratio.

Interviews were conducted by telephone approximately eight days after recruitment and every six months subsequently. Participants received a $50 gift card after completing each interview. The study is ongoing; participants are being followed for five years. This analysis uses data from the first thirty months, or six interviews. The Turnaway Study was approved by the Committee for Human Research at the University of California, San Francisco.

### Measures

#### Outcomes

*Perceived stress* was measured using a modified version of the four-item Perceived Stress Scale (PSS) scale [1]. Each PSS item assesses the frequency with which respondents felt overwhelmed or able to cope in the past month, e.g. “how often have you felt difficulties were piling up so high that you could not overcome them?” (never to very often, on a 5-point scale). For this study, items were modified (PSS-M) to ask about the degree to which these items were felt in the last seven days (not at all to extremely, on a 5-point scale). Stress over the last week (as opposed the last month) was asked in order to correspond with the period between receiving or being denied the abortion and the first interview. The scale developers state that the scale should be as reliable over a shorter period of time as it is over one month. Positive items were reverse coded, and item responses were summed to form a 0-16 scale in which higher scores represent more stress. The PSS-M had a one-component structure and a Cronbach’s α of 0.74.

Our measure of *emotional social support* was based on the twelve-item Multidimensional Scale of Perceived Social Support (MSPSS) [43]. The scale includes items about the perceived availability of emotional support from friends, family, and significant others (e.g. “I can talk about problems with my friends”). The modified version used in this study (MSPSS-M) retained two items for each of the three support sources. Response categories for the MSPSS are a seven point Likert scale (very strongly disagree to very strongly agree); this study used a five point scale (strongly disagree to strongly agree). Following MSPSS scoring, item responses were summed, and the scale was scored as a mean of the six items. Higher scores indicated higher social support. The MSPSS-M had a one-component structure and a Cronbach’s α of 0.80.

Both outcome measures asked about women’s lives in general, and did not specifically refer to the experience of seeking an abortion.

#### Independent variables

The primary independent variables of interest were *time* (months since recruitment) and *study group,* as described in the methods section*.* For analysis, the Turnaway Group was divided into Parenting Turnaways – women who gave birth after being denied abortion and did not place the baby for adoption, and Non-parenting Turnaways – women who received an abortion elsewhere, had a miscarriage, or placed their baby for adoption. The Non-parenting Turnaway group was included in analyses for proper calculation of site variance; however, results for the group are not presented due to the diverse range of experiences of women in the group. The Near Limit Abortion Group served as the reference group for analyses to permit direct and simultaneous comparisons with both the Parenting Turnaway and First Trimester Groups.

Demographic, mental health and stressor baseline variables were considered for inclusion as model covariates if there was theoretical basis or prior evidence for association with study group and the outcome of interest.

Demographic variables included *age*, *race*, *household composition*, *parity*, *maternal education level,* and *school/working status*. For the household composition variable, women who lived with more than one other adult were assigned to categories in the following order: any male partner, any adult relative, other non-related adults or no other adults. Marital status was not included due to high correlation with household composition. Maternal education and school/working status are indicators of socioeconomic status.

Mental health history variables included report of previous clinical diagnosis of *anxiety*, and report of previous clinical diagnosis of *depression*.

Five stressor variables included *experience of abuse or neglect before age 18*; *experience of violence in the past year* (physical or psychological); *poor self-rated pre-pregnancy physical health*, coded as yes if fair, poor or very poor and no if good or very good; *current chronic pain* (at least one type among abdominal, pelvic, lower back, arthritis, head, and other); and *current caretaker to sick relative*. An additional composite variable, *stressful life* events was defined as reporting being quite a bit or extremely affected by at least one of seven stressful life events in the past (physical attack/assault, life-threatening accident, house robbed, serious illness or injury of self or of partner, mental illness in household during childhood, drinking or drug problems in household during childhood).

Demographic, mental health history, and stressor variables were considered as covariates for the perceived stress model. The mental health and stressor variables were considered for inclusion in this model to help account for prior stress because there was no measure of perceived stress before the participants sought abortion. Demographic variables were considered as covariates for the social support model. We adjusted for covariates in our models to remove confounding due to imbalance in these covariates by study group at baseline. Only covariates that differed significantly between groups at baseline were included in the models. Because the study was not designed to assess the relationship between the covariates and outcomes, we do not discuss covariate estimates.

### Statistical analysis

Data were analyzed with STATA12. Associations between baseline covariates and study group were assessed using mixed effects regression models with random intercepts for recruitment facility to account for clustering by site. Covariates that differed between the study groups at P < 0.10 were retained for longitudinal models to control for baseline differences.

To assess trends in each outcome over time by study group, a longitudinal mixed effects model was constructed with random intercepts for site and participant, and random slopes for time if inclusion of the random slope improved model fit as determined by a likelihood ratio test. This type of model accounts for loss to follow up over time, using information from individuals’ observed data to inform the unobserved data [44]. Quadratic and cubic terms for time were added if they improved model fit, also determined by a likelihood ratio test. These terms were considered because pregnancy, birth and abortion are complex psychosocial periods in women’s lives, and quadratic or cubic terms might be necessary to capture trends in responses over time. Sensitivity analyses were performed restricting the population to participants at sites with recruitment rates of 60% or higher.

## Results

### Retention and follow up

Among eligible women approached, 37.5% consented to participate in the five year study. Participants from one facility at which 90% of Turnaways obtained an abortion elsewhere were excluded from analysis (n = 76). The final sample included 413 Near Limit Abortion participants, 254 First Trimester participants, 146 Parenting Turnaway participants and 64 Non-parenting Turnaway participants. The Non-parenting Turnaway participants included 44 who received an abortion elsewhere, 15 who placed the baby for adoption, and five who had a miscarriage or stillbirth. Participant retention at 6 and 30 months was 92% and 72%, respectively. Retention did not significantly differ by study group or by baseline perceived stress or social support.

### Baseline characteristics

The Parenting Turnaway Group was on average slightly younger (23.4 years) and the First Trimester Abortion Group slightly older (25.9) than the Near Limit Abortion Group (24.9) (Table 1). By study design, gestational age at recruitment differed significantly by study group (P < 0.001). The groups had significant differences in history of diagnosed depression (25% in the First Trimester Group vs. 19% in the Near Limit Abortion Group, P < 0.10) but did not differ significantly in history of anxiety. The groups also had significant differences in history of child abuse/neglect (17% among Non-parenting Turnaways compared with 26% in the Near Limit Abortion Group, P < 0.10, results not shown) and experience of violence in past year (16% among Parenting Turnaways compared with 23% in the Near Limit Abortion Group, P < 0.10), but no differences in other stressors.

**Table 1:** Baseline characteristics of participants by study group

	**Near limit abortion (ref) n = 413**		**First trimester abortion**^**a**^**n = 254**		**Parenting turnaway**^**a**^**n = 146**	
	**%**	***n***	**%**	***n***	**%**	***n***
**Demographics**						
Age (mean, *SD*)^b^ (n = 813)	24.9	*5.9*	25.9*	*5.7*	23.4**	*5.5*
Gestational age in weeks (mean, *SD*) (n = 813)	19.7	*4.1*	7.6***	*2.3*	23.1***	*3.4*
Race/ethnicity (n = 813)			*			
*Ehite*	32	*132*	39	*99*	24	*35*
*Black*	32	*131*	32	*80*	34	*50*
*Hispanic/Latina*	21	*87*	21	*54*	29	*42*
*Other*	15	*63*	8	*21*	13	*19*
Household composition (n = 813)			*		*	
*No other adult*	30	*122*	36	*92*	20	*29*
*Male partner*	24	*99*	30	*77*	20	*29*
*Adult relative*	38	*255*	26	*66*	49	*71*
*Non-adult relative*	9	*37*	8	*20*	12	*17*
Parity (n = 813)					**	
*Nulliparous*	33	*604*	35	*403*	48	*303*
*Baby under 1*	12	*228*	11	*126*	6	*39*
*1+ previous births, no new baby*	25	*432*	20	*213*	17	*116*
*2+ previous births, no new baby*	30	*537*	34	*373*	29	*176*
Mother's Highest Level of Education (n = 813)			**			
*<high school*	12	*51*	20	*52*	14	*21*
*High school or GED*	36	*148*	36	*91*	35	*51*
*Associate’s, some college, tech school*	19	*80*	14	*35*	21	*30*
*College*	22	*92*	24	*62*	19	*28*
*Missing*	10	*42*	6	*14*	11	*16*
School/working status (n = 813)			*		+	
*Not in school or employed*	32	*132*	23	*58*	38	*55*
*In school*	14	*57*	13	*34*	21	*30*
*Employed*	40	*167*	42	*105*	30	*44*
*In school and employed*	14	*57*	22	*56*	12	*17*
**Mental health** (n = 813)						
History of depression	19	*77*	25+	*63*	15	*22*
History of anxiety	15	*62*	16	*41*	12	*18*
**Stressors** (n = 813)						
History of child abuse/neglect	26	*108*	28	*71*	25	*37*
Experienced violence in past year	23	95	24	60	16+	23
Was strongly affected by at least one stressful life event	33	*138*	36	*92*	30	*44*
Caretaker to sick relative	7	*30*	5	*12*	9	*13*
Has any type of chronic pain	34	*139*	39	*99*	39	*57*
Poor self-rated physical health	18	*76*	20	*51*	17	*25*

^a^group differs from Near Limit Abortion Group by + p < .10, *p < .05, **p < .01, ***p < .001.

^b^Sample includes one participant aged 14 who was recruited early in the study before the minimum enrollment age was changed to 15. Ref = reference group, SD = standard devision.

### Perceived stress

**Figure 1:**
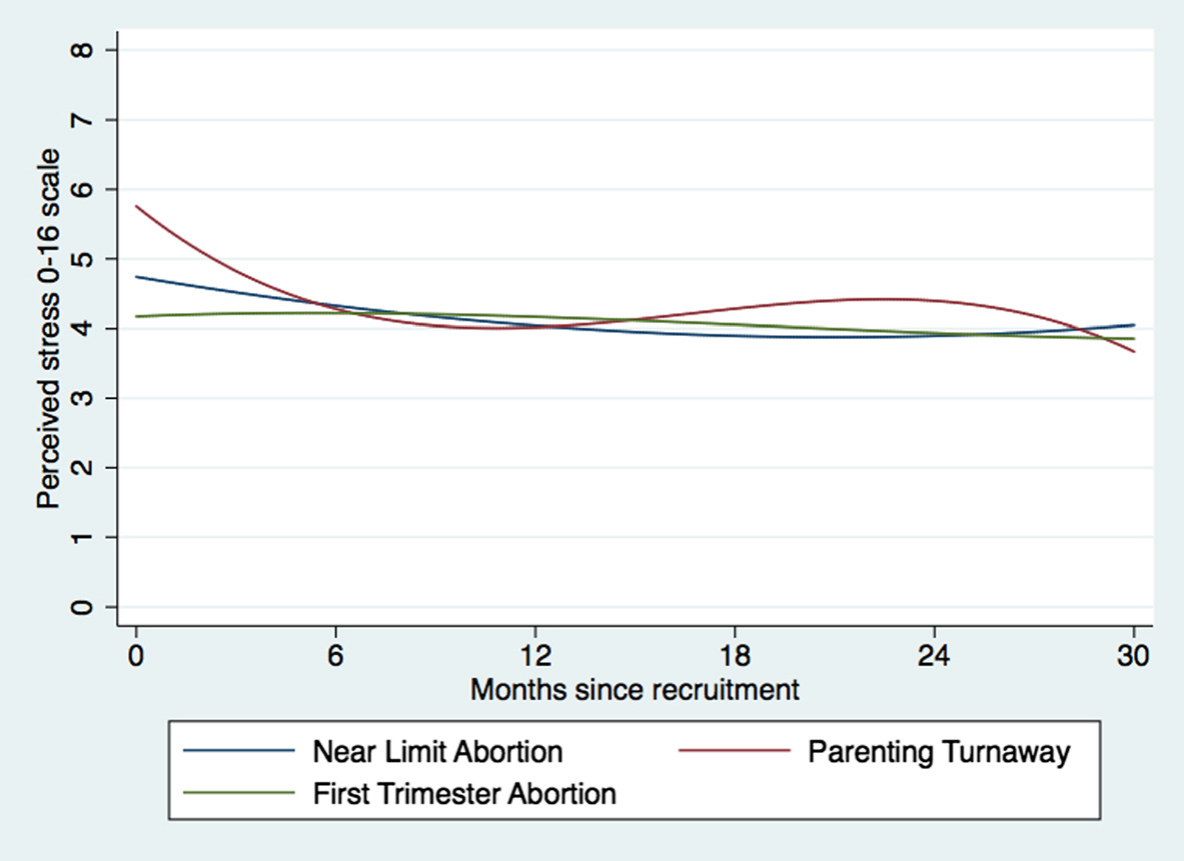
Multivariate mixed effects model of perceived stress by study group over time.

Table 2 and Figure 1 show the results of an adjusted longitudinal model of perceived stress by study groups. The coefficients for time, time squared and time cubed refer to the change over time of the reference Near Limit Abortion Group; the coefficients for interaction terms between time and other study groups refer to how change over time for the respective study group differs from the Near Limit Abortion Group. During the week between receiving the abortion and completing the baseline interview, the Near Limit Abortion Group had a stress score of 4.7 on the 0-16 scale, holding covariates constant (model constant in Table 2). Over the week after being denied an abortion, perceived stress for the Parenting Turnaway Group was on average one unit higher compared to the Near Limit Abortion Group (B = 1.02, P = 0.003). 2Perceived stress was half a unit lower for the First Trimester Group than for the Near Limit Group (B = -0.57, P = 0.045) at one week. As shown in Figure 1 and as indicated by the significant months term, the Near Limit Abortion Group’s perceived stress levels decreased over time, with the First Trimester Group experiencing similar changes. Reductions in stress were greater among the Parenting Turnaways, which converged with the Near Limit group by 6 months and remained similar through 30 months. Based on Figure 1, stress decreased slightly or remained steady for all groups during this period.

**Table 2:** Multivariate mixed effects model of perceived stress by study group over time

	**Coefficient**	**95% conf. interval**	
**Study group**			
Near Limit Abortion Group	ref	ref	ref
First Trimester Abortion Group	-0.57*	-1.13	-0.01
Parenting Turnaway Group	1.02**	0.34	1.69
**Time**			
Months	-0.08	-0.16	0.00
Months^2^	0.002	-0.005	0.008
Months^3^	0.000	0.000	0.000
**Study group by time interactions**			
First trimester x months	0.10	-0.03	0.23
First trimester x months^2^	-0.004	-0.014	0.006
First trimester x months^3^	0.000	0.000	0.000
Parenting turnaway x months	-0.31***	-0.46	-0.15
Parenting turnaway x months^2^	0.02***	0.01	0.04
Parenting turnaway x months^3^	-0.001***	-0.001	0.000
**Covariates**			
Age	0.126	-0.01	0.06
Race/ethnicity			
*White*	ref	ref	ref
*Black*	0.114	-0.08	0.80
*Hispanic/Latina*	0.660	-0.38	0.61
*Other*	0.666	-0.68	0.44
Household composition			
*No other adult*	ref	ref	ref
*Male partner*	0.041	-0.93	-0.02
*Adult relative*	0.197	-0.77	0.16
*Non-adult relative*	0.099	-1.16	0.10
Parity			
*Nulliparous*	ref	ref	ref
*Baby under 1*	0.036	0.04	1.23
*1+ previous births, no new baby*	0.353	-0.24	0.68
*2+ previous births, no new baby*	0.900	-0.52	0.45
Mother's highest level of education			
*<high school*	ref	ref	ref
*High school or GED*	0.443	-0.72	0.31
*Associate’s, some college, tech school*	0.905	-0.62	0.55
*College*	0.098	-1.03	0.09
*Missing*	0.942	-0.68	0.73
School/working status			
*Not in school or employed*	ref	ref	ref
*In school*	0.019	-1.18	-0.11
*Employed*	<0.001	-1.14	-0.32
*In school and employed*	0.020	-1.17	-0.10
History of depression	<0.001	1.29	2.15
Experienced violence in past year	0.001	0.30	1.13
History of child abuse/neglect	0.001	0.25	1.04
Model constant	4.74***	4.09	5.39

*p < .05, **p < .01, ***p < .001. ref = reference group, SD = standard deviation.

### Emotional social support

**Figure 2:**
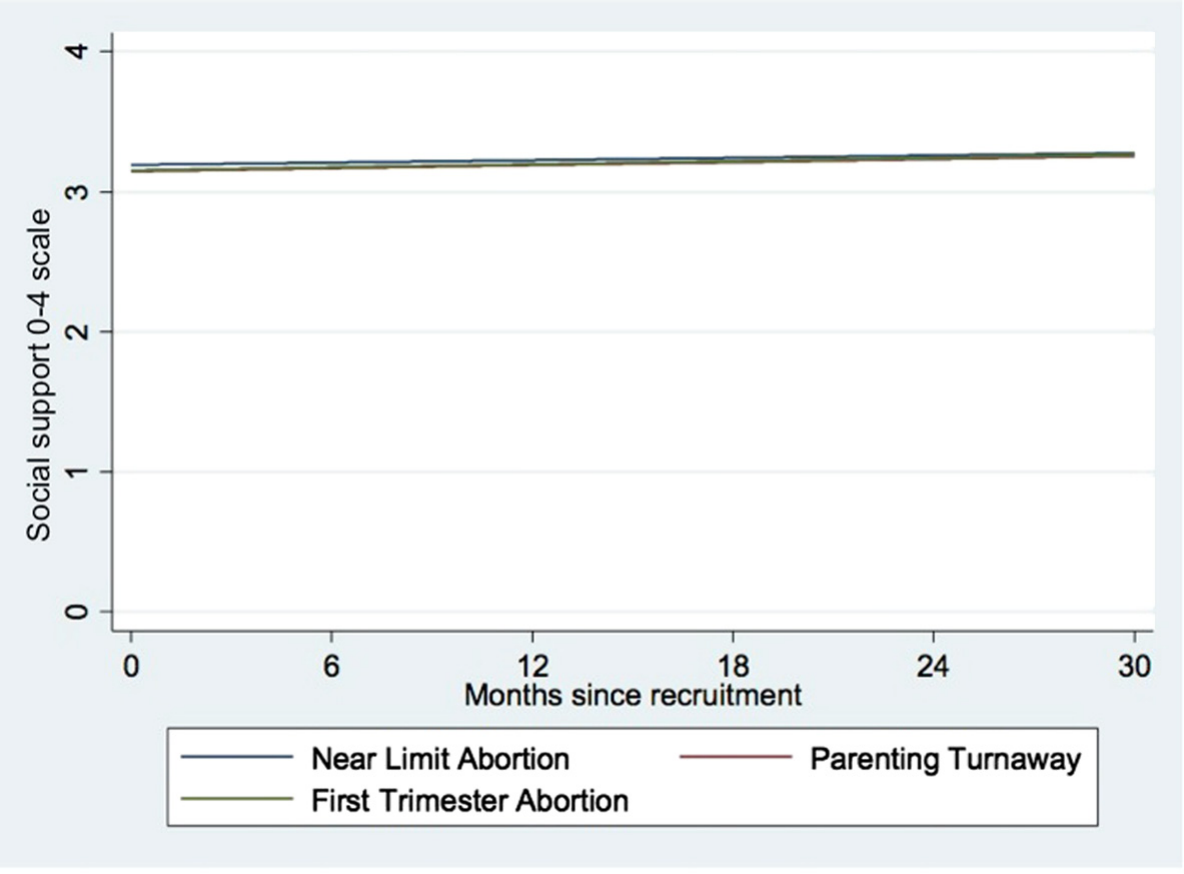
Multivariate mixed effects model of social support by study group over time.

Social support did not differ significantly by study group at one week or over time (Table 3 and Figure 2). At one week, all study groups reported an average of 3.2 units of social support on the 0-4 scale, holding covariates constant (model constant in Table 3). Levels of social support rose very gradually over time (0.08 units – 2% of the total scale – over 30 months, P = 0.05) for all groups, with no differences in increase by group.

**Table 3:** Multivariate mixed-effects model of social support by study group over time

	**Coefficient**	**95% conf. interval**	
**Study group**			
Near Limit Abortion Group	ref	ref	ref
First Trimester Abortion Group	-0.04	-0.13	0.05
Parenting Turnaway Group	-0.05	-0.16	0.06
**Time**			
Months	0.003**	0.001	0.005
**Study group by time interactions**			
First trimester x months	0.001	-0.002	0.004
Parenting turnaway x months	0.001	-0.003	0.005
**Covariates**			
Age	0.00	-0.01	0.00
Race/ethnicity			
*White*	ref	ref	ref
*Black*	-0.21***	-0.30	-0.12
*Hispanic/Latina*	-0.20***	-0.30	-0.10
*Other*	-0.09	-0.20	0.03
Household composition			
*No other adult*	ref	ref	ref
*Male partner*	0.05	-0.04	0.15
*Adult relative*	0.08	-0.02	0.18
*Non-adult relative*	0.02	-0.11	0.16
Parity			
*Nulliparous*	ref	ref	ref
*Baby under 1*	-0.08	-0.20	0.05
*1+ previous births, no new baby*	-0.17*	-0.26	-0.07
*2+ previous births, no new baby*	-0.13	-0.23	-0.03
Mother's highest level of education			
*<high school*	ref	ref	ref
*High school or GED*	0.15**	0.04	0.25
*Associate’s, some college, tech school*	0.12	0.00	0.24
*College*	0.24	0.13	0.36
*Missing*	-0.09	-0.24	0.06
In school/working			
*Not in school or employed*	ref	ref	ref
*In school*	0.21***	0.10	0.32
*Employed*	0.22***	0.14	0.30
*In school and employed*	0.23***	0.12	0.33
Model constant	3.19***	3.07	3.32

*p < .05, **p < .01, ***p < .001, ref = reference group, SD = standard deviation.

### Sensitivity analyses

Results were similar when restricting analysis to study sites with a recruitment rate of at least 60% (7 sites, N = 413). Perceived stress followed the same initial pattern of being highest among Parenting Turnaways, less high in women who received near limit abortions, and lowest among women with first trimester abortions – although the difference between stress levels among Parenting Turnaways and the Near Limit Abortion Group was no longer significant at P < 0.05. Over time, perceived stress levels in each group were similar to those of the full analysis in shape and magnitude. Results for social support were unchanged.

## Discussion

This study found that in the week after receiving or being denied an abortion, women who were denied abortions experienced higher stress compared to women who received abortions, and that women who received later abortions had higher stress initially than women receiving first trimester abortions. However, perceived stress levels for all three groups converged by six months and decreased similarly over the follow-up period of 30 months. The study also found that initial levels of social support and trends over time did not differ significantly between groups during the 30 months after seeking abortion. Social support increased for all groups over this period; the magnitude of this increase was small, at 2% of the total scale.

### Perceived stress

Our finding that women denied abortions were initially more stressed than women receiving abortions was expected. Unwanted pregnancy can be a stressful experience for any woman [22], but being denied an abortion may exacerbate stress, making a woman feel disempowered or unable to cope with the stressor [11,45]. Preparing to carry an unwanted pregnancy to term, managing pregnancy itself, and potentially searching for another abortion provider may also contribute to stress [45].

The finding that women who received later abortions were initially more stressed than women who received first trimester abortions may be a consequence of different factors that caused the delay in seeking abortion and that were unobserved in our data. Delayed abortion-seeking is associated with factors that may be associated with stress, such as greater indecision and fear of abortion as well as socioeconomic and logistical barriers [25,46,47]. Women who receive later abortions may also be subject to greater stigma because they may be unable to hide pregnancies. Further, they may experience more stress from the process of arranging and receiving a later abortion, and this stress may persist after the denial of abortion. Importantly, the differences in stress levels between women having later versus first trimester abortions were not sustained over time.

Previous research on stress and pregnancy provides little insight into the outcomes after unwanted pregnancy over time, as few studies compare parents and non-parents [22] and most do not consider the wantedness of the pregnancy [48]. However, early motherhood is a stressful period [49], perhaps more so if the baby is the result of an unwanted pregnancy [21]. That Parenting Turnaways experienced decreasing stress over time may be surprising and suggests that women are resilient to this pregnancy outcome. This result adds to the literature on pregnancy outcomes and resiliency, which shows that although women’s experiences of pregnancy and childbearing can have long term outcomes for mothers and children, women are also resilient in many ways to the challenges faced during these periods [50,51].

This analysis adds to the literature on abortion and psychosocial well-being by finding slight decreases in perceived stress over time after an abortion. This finding is particularly important given the narrative that abortion is stressful for women and harms women’s psychosocial well-being [7], which has gained traction popularly and politically but is not supported by rigorous research [14,24]. Other work from the Turnaway study has similarly found that the experience of abortion does not result in mental health harm [49].

Mean perceived stress scores for each study group (initially between 4-6 on a 0-16 scale) appear comparable to or lower than previous literature on perceived stress in national samples of women [2]. Importantly, stress did not increase beyond initial levels for any group over the 30 months.

### Social support

Emotional social support did not differ by group at one week or over time. Social support is critical to overall health and well-being, particularly when coping with challenging life events [19,34,52]. We had expected to find a differential in social support because abortion can be highly stigmatized in the US and may lead to loss of social support for some women [53], while Parenting Turnaways may have received social support to help them meet the demands of motherhood. One explanation for the lack of difference in social support between women who have abortions and who carry the pregnancy to term is that unwanted birth is as stigmatized as abortion and hence the lack of difference. This explanation is not wholly satisfying given that on average, all groups report an increase in social support over time. Another explanation for the similar group trajectories is that our measure detected perceptions of the adequacy of social support; if women’s need for emotional support differs by group, this may obscure differences in actual social support. Further, our measure is of emotional support rather than practical, logistical or financial social support; we cannot detect changes in these other types of social support although they are important to the period surrounding pregnancy [35,43]. Finally, the MSPSS measures social support in general rather than related to a specific life event; items may not have captured the nuances of emotional social support in the context of pregnancy and abortion or childbearing [43].

### Strengths and limitations

This is the first known analysis that compares perceived stress or social support among women who received abortions to women who sought but did not receive an abortion. The Turnaway Study design overcomes many of the methodological challenges common to research on the effects of abortion: it has longitudinal follow-up, uses a comparison group that represents what women’s experience would have been had they not terminated the unwanted pregnancy, and does not suffer from underreporting of abortions [9,10].

A potential limitation of this study is the 37.5% rate of participation among those approached, likely a consequence of asking women to participate in a study for five years, and to discuss a stigmatized topic. Variation in participation rates (from less than 30% to 80%) was observed by site. Findings were generally unchanged in analyses among higher participation sites only, supporting the validity of results among all participants. Women in the sample had similar demographic characteristics to a representative sample of US women with unintended pregnancies [54]. Women in the study who received abortions were similar demographically to women receiving abortions in the U.S. with one exception: women in the study had higher rates of poverty.

A final limitation is that we could not assess whether study groups had similar levels of stress and social support prior to seeking abortion. Ideally, we would have controlled for prior stress and social support, but asking participants to recall perceived stress and emotional social support prior to seeking abortion would have been vulnerable to recall bias. To address this limitation, we controlled for key confounders such as prior mental health problems, stressors, and household composition. Further, because these data did not permit an assessment of social support and stress before seeking abortion, it is possible that the time at which these women started the study represented a peak in stress or low-point in social support, which would help explain why they both improved over time.

Another limitation is that psychometric scales were modified. The lead-ins to items and answer choices were changed to match with lead-ins and answer choices for other questions in the interview, but the items themselves were identical to the original. However, both modified scales had good internal consistency and represented a single factor in this study.

### Implications

Perceived stress levels were highest shortly after seeking abortion, and higher among Turnaways and women who received second trimester abortions than among first-trimester abortions. Further, immediate stress was highest among women who were denied abortions. Counseling is usually geared towards women receiving abortions [34,38], and little is known about the support provided to women who are turned away. Women who are turned away face unique challenges – many will go on to parent, and others must deal with obtaining an abortion elsewhere or placing the child for adoption. Interventions should focus on developing ways to support women who are denied wanted abortions, including appropriate referrals to abortion providers who can accommodate their gestational age, and how to identify and meet their unique needs.

In addition, given the negative effects of stress during pregnancy on maternal and child health, it is important to consider how new laws restricting access to abortion may affect the health and well-being of women and their children. Even though the elevation of stress among Turnaways was temporary, stress was elevated during a period that can have long term mental and physical health outcomes for mothers and children [17-20].

This study also adds to a growing body of literature showing that receiving an abortion does not result in worse psychosocial outcomes than carrying an unwanted pregnancy to term. Thus, it provides no support for the notion that abortion hurts women’s well-bring, or for policies that restrict abortion or mandate counseling based on this notion.

## Conclusion

In this study, neither receiving nor being denied an abortion resulted in increasing stress or loss of social support over 30 months. Efforts should focus on providing counseling and support to women who are denied abortions due to gestational age limits to help support them through the stress they may experience initially.

## Abbreviations

MSPSS: Multidimensional scale of perceived social support; MSPSS-M: Multidimensional scale of perceived social support, modified version; PSS: Perceived stress scale; PSS-M: Perceived stress scale, modified version; US: United States.

## Competing interests

The authors declare that they have no competing interests.

## Authors’ contributions

DF designed and led the Turnaway study. LH and DF designed the analysis. LH analyzed the data with input from SR, MAB, and CR. LH wrote the manuscript; SR, MAB, CR and DF substantially revised manuscript for intellectual content. All authors read and approved the final manuscript.

## Pre-publication history

The pre-publication history for this paper can be accessed here:

http://www.biomedcentral.com/1472-6874/14/76/prepub
